# Network-driven design principles for neuromorphic systems

**DOI:** 10.3389/fnins.2015.00386

**Published:** 2015-10-20

**Authors:** Johannes Partzsch, Rene Schüffny

**Affiliations:** Chair for Highly Parallel VLSI Systems and Neuromorphic Circuits, Department of Electrical Engineering and Information Technology, Technische Universität DresdenDresden, Germany

**Keywords:** neuromorphic architectures, synaptic connectivity, system design, Rent's rule, mapping quality

## Abstract

Synaptic connectivity is typically the most resource-demanding part of neuromorphic systems. Commonly, the architecture of these systems is chosen mainly on technical considerations. As a consequence, the potential for optimization arising from the inherent constraints of connectivity models is left unused. In this article, we develop an alternative, network-driven approach to neuromorphic architecture design. We describe methods to analyse performance of existing neuromorphic architectures in emulating certain connectivity models. Furthermore, we show step-by-step how to derive a neuromorphic architecture from a given connectivity model. For this, we introduce a generalized description for architectures with a synapse matrix, which takes into account shared use of circuit components for reducing total silicon area. Architectures designed with this approach are fitted to a connectivity model, essentially adapting to its connection density. They are guaranteeing faithful reproduction of the model on chip, while requiring less total silicon area. In total, our methods allow designers to implement more area-efficient neuromorphic systems and verify usability of the connectivity resources in these systems.

## 1. Introduction

With neuromorphic systems growing in size (Schemmel et al., 2010; Benjamin et al., 2014; Furber et al., 2014; Merolla et al., 2014b), efficient realization of synaptic connectivity becomes an ever more important part of the design. Sufficiently many configurable synaptic connections are crucial for applicability of these systems, while typically dominating overall silicon area. Thus, the main challenge is to decrease the mean area per synapse, while retaining enough flexibility to be able to map all relevant application-specific connectivity models onto the hardware.

Several chip architectures and implementation approaches have been proposed, spanning a wide range of this trade-off between flexibility and area consumption. Classic architectures utilize a synapse matrix, differing mainly in their access to single synapses. Synapses may be addressed individually using xy-decoders, as employed e.g., in Chicca et al. (2004) and in the FLANN chip (Giulioni et al., 2008). Alternatively, they may be accessed column-wise, forming a crossbar architecture, as implemented in the Spikey (Schemmel et al., 2006) and TrueNorth (Merolla et al., 2014b) systems. Additional source selection may be stored in individual synapses, as done in the BrainScaleS waferscale system (Schemmel et al., 2010) and in the MAPLE chip (Noack et al., 2010). Qiao et al. (2015) employ the same principle to switch between individual and column-wise access, combining advantages of both architectures. As an alternative to synapse matrices, neuron arrays have been implemented (Choi et al., 2004; Yu et al., 2012), being especially suited for nearest-neighbor synaptic connectivity. A more fine-grained control on individual neuron structure is gained by field-programmable neural arrays (FPNA) (Farquhar et al., 2006), enabling to mimick detailed dendritic structure. In a more high-level approach, neuron and synapse models may be emulated on general-purpose processors, as done in the SpiNNaker system (Furber et al., 2014), trading higher flexibility against potentially less energy efficiency.

Another common option is to utilize multi-synapse circuits, which allow to calculate the joint effect of multiple synapses in one circuit. This approach was used for example by Vogelstein et al. (2007) and in the NeuroGrid system (Benjamin et al., 2014). Multi-synapse circuits typically do not include storage of synaptic weights, but weight values are stored separately, often off-chip in an FPGA. This is a fundamental difference to systems with a synapse matrix, where each synapse circuit performs both weight storage and weight effect calculation. Also, long-term learning, i.e., modification of synaptic weights, is not included in the multi-synapse circuit, but implemented separately. As a consequence, systems with multi-synapses often allow to integrate significantly more neurons per chip, by removing the area-intensive synaptic weight storage and weight modification circuitry from the chip. This, however, comes at the price of more complexity off-chip, e.g., implementing the latter functions in an FPGA. Therefore, meaningful comparisons to other approaches can only be made on system level.

While the decision for one of the above implementation approaches is often model-driven, the design process typically focuses on technical aspects, choosing element count and configurability mainly on the overall area budget. Several works investigate architectures concerning their technical complexity, either on chip level (Benjamin et al., 2014) or inter-chip level (Culurciello and Andreou, 2003; Park et al., 2012; Merolla et al., 2014a). In contrast, verification and optimization of the chip architecture with respect to connectivity models is done only in a later stage of the design (Fieres et al., 2008). After implementation, several works optimize neuron placement and connection routing for improving mapping of specific networks to the finished system (Navaridas et al., 2009; Brüderle et al., 2011). While these works shed some light into the relationship between connectivity models and neuromorphic architectures, they provide only incomplete guidance during design. A systematic method for architecture design is missing, which would allow to tailor the architecture to a set of given connectivity models, utilizing the models' constraints for optimization, for example reducing area and power consumption.

In this paper, we provide first steps toward such a method. We demonstrate how to use Rent's Rule (Landman and Russo, 1971; Christie and Stroobandt, 2000; Partzsch and Schüffny, 2012) for characterizing synaptic connectivity. This tool allows to extract a specification for the amount of connectivity in a hierarchical neuromorphic system. Having derived the required number of synapses and inputs for a single chip with this method, we move to designing the chip architecture. For this, we introduce a generalized synapse matrix architecture that unifies description of state-of-the-art designs. This architecture inherently enables shared use of circuit components for minimizing total silicon area. We demonstrate how to parameterize the generalized architecture such that it faithfully reproduces a given connectivity model, adapting the architecture to the model's local connection density. Finally, we show how to find the most area-efficient architecture dependent on the sizes of individual circuit components. The whole set of methods enables a fully top-down approach, guaranteeing faithful reproduction of a connectivity model and providing an informed decision about the most area-efficient architecture for a given use case.

The article is structured as follows: Sections 2.1 and 2.2 classify existing neuromorphic architectures. Sections 2.3 and 2.4 introduce the generalized synapse matrix architecture and show how to evaluate it. Rent's Rule is described in Section 2.5. Section 3.1 characterizes two existing architectures with the introduced methods. Sections 3.2 and 3.3 introduce the top-down design approach, with a special case handled in Section 3.4. Finally, Section 3.5 provides an architecture comparison concerning total silicon area.

## 2. Materials and methods

### 2.1. Classification of model components

**Figure 1:**
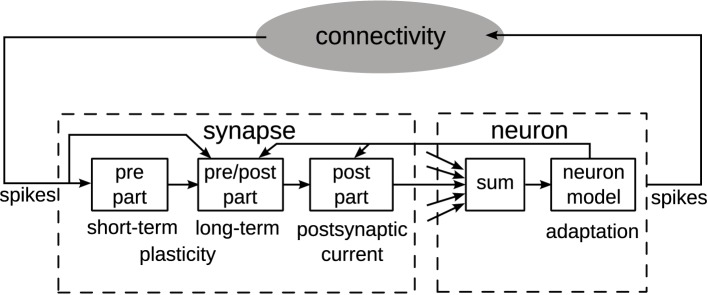
**Separation of synapse and neuron model components into parts and data dependencies (Partzsch, 2014)**. A single synapse and neuron are shown. Multiple synapses connect to a neuron, which is indicated by the arrows to the summation block. From this single-element view, neuromorphic system architectures fall within the black-box module “connectivity.”

In an abstract view, neuron and synapse models may be separated into parts with differing data dependencies, which directly influences the choice of an architecture (Benjamin et al., 2014). Figure 1 shows one such partitioning, assuming point neurons (Gerstner and Kistler, 2002).

The synapse model may be split into a pre-synaptic, a post-synaptic, and a combined pre-synaptic–post-synaptic (pre/post) part. The pre-synaptic part contains all model components that are only dependent on input spikes. In consequence, it can be shared by all synapses with the same pre-synaptic input. Short-term plasticity models (Markram et al., 1998) typically fall within this part. In turn, the post-synaptic part contains all model components that are only dependent on variables from the connected post-synaptic neuron. This part may be implemented only once for all synapses of that neuron. Generation of exponentially decaying post-synaptic currents or conductances is a typical example of this part (Schemmel et al., 2010). Only the pre/post part is individual to each synapse, typically containing the synaptic weight and some form of long-term plasticity (Mayr and Partzsch, 2010; Azghadi et al., 2014). Often, long-term plasticity models themselves can be separated in pre- and post-synaptic parts, implementing traces of pre- and post-synaptic activity, and a pre/post part that combines these traces. This separation reduces complexity in single synapses (Mayr et al., 2010). It also enables plasticity implementation possible in memristive crossbars, reducing single memristive elements to integrators of differences between pre- and post-synaptic voltage traces (Mayr et al., 2012; Saighi et al., 2015).

The neuron typically consists of a summation over all connected synapses and the neuron model itself, possibly including some form of adaptation (Naud et al., 2008). The output spikes of the neuron are transmitted via some connection fabric on- and off-chip to the targeted synapses. Properties of the connection fabric are greatly influenced by the arrangement of synapses and neurons into chip architectures, which are introduced in the next section.

### 2.2. State-of-the-art neuromorphic chip architectures

The diversity of existing neuromorphic chip architectures is high, reflecting the different approaches to implementing neurons and synapses, as was discussed in the Introduction. For this article, we restrict ourselves on architectures with a synapse matrix, implementing an individual circuit for each synapse in the network.

Using a synapse matrix may seem inefficient and inflexible at first sight, given alternatives such as multi-synapses or multi-processor systems. However, synapse matrices are advantageous for long-term synaptic learning, which is regarded as an essential part of neural processing (Azghadi et al., 2014), while typically dominating computational effort of the system, because calculations have to be carried out individually for each synapse. In Figure 1, this is reflected by the pre/post part of the synapse, whose data dependencies prevent joint calculations between synapses. As a consequence, long-term learning can not be implemented with multi-synapses, which represent joint synaptic activity in a single circuit. While multi-synapses allow for an efficient separation between forward operation of a neural network, i.e., calculating the expression of synaptic weights, and storage of synaptic weights, for example in an area-efficient external dynamic random-access memory (DRAM), long-term learning would require separate circuitry. Multi-processor systems allow for implementing almost arbitrary synaptic learning rules, at the expense of reading and writing synaptic weights to an external RAM at each weight update. This read/write procedure constitutes a potential bottleneck in terms of throughput, and it puts a lower limit on the achievable energy efficiency, given by the energy to communicate a synaptic weight between processor and RAM. In comparison, synapse matrix architectures at least conceptually offer a significantly more energy-efficient solution by performing calculations on synaptic weights as close as possible to their storage, combining processing and storage in the individual synapse circuit.

**Figure 2:**
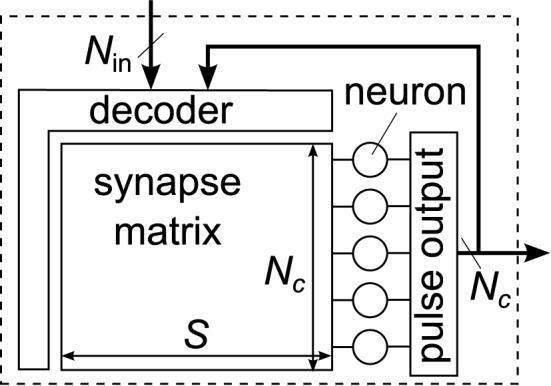
**General structure of a neuromorphic chip or block with synapse matrix**. A decoder forwards inputs and locally fed-back neuron outputs to the synapses in the matrix. Each neuron has the same number of synapses *S*, allowing for the matrix layout.

The general structure of a neuromorphic chip or block with synapse matrix is shown in Figure 2. It can be characterized by the number of neurons *N*_*c*_, the number of synapses per neuron *S*, and the number of inputs to the block, *N*_in_. The number of synapses, calculated as *N*_*c*_·*S*, typically dominates the total silicon area and thus often limits the size of the block. From the viewpoint of Figure 2, differences in architectures mainly arise from the type of input decoder, forwarding inputs to selected synapses.

When using an xy-decoder, a single synapse is activated for each input spike, using a column and row enable line (Chicca et al., 2004; Giulioni et al., 2008). This architecture gives the most flexible control over synaptic connectivity, and is therefore named fully addressable matrix in the following. As a downside of the flexible access, no sharing of the pre-synaptic circuit part between synapses is possible, because each synapse may receive a different pre-synaptic input (cf. Figure 1). Also, the decoder does not realize any fan-out, so that one spike packet has to be transmitted for each synaptic connection, resulting in higher input packet rate compared to the other architectures.

In a crossbar architecture, each input spike drives a complete synapse column (Schemmel et al., 2006; Merolla et al., 2014b). Compared to a fully addressable matrix, the number of inputs is lowered by a factor of *N*_*c*_ for the same matrix size, accompanied by a significant reduction in flexibility, as we show in Section 3.1. However, all synapses of one column share the same pre-synaptic input, so that the pre-synaptic part of the synapse has to be implemented only once per column. Also, as one input fans out to the whole column, part of the synaptic fan-out is realized by the crossbar itself, which significantly reduces input bandwidth.

Other architectures with synapse matrix constitute intermediate solutions between fully addressable matrix and crossbar. The BrainScaleS waferscale system described in Schemmel et al. (2010) equips each synapse with a 4 bit source configuration, allowing it to select from 16 inputs. This strategy still allows to partially share circuitry among synapses, while gaining significantly more flexibility compared to a crossbar architecture. Further input selection is performed at the side of the synapse matrix and via switchable routing channels on-chip. Additionally, neighboring neuron circuits may be connected together, forming neurons with more synapses.

The implementation by Qiao et al. (2015) demonstrates how to overlay a fully addressable matrix and a crossbar architecture by adding a 1 bit configuration in each synapse. With this extension, all activated synapses in one column may be triggered by a single input spike, while the other synapses may still be accessed individually as in the fully addressable matrix. This reduces input bandwidth, while keeping the architecture flexible. Like for the BrainScaleS system, neuron circuits may be joined for realizing neurons with higher synapse count.

Another architecture was implemented in the MAPLE chip (Noack et al., 2010; Mayr et al., 2013). In this design, pre-synaptic driver circuits are placed on both sides of the synapse matrix. Each synapse contains 1 bit input configuration, letting it choose from one of two driver circuits at the two sides. This doubles the number of inputs to the matrix compared to a crossbar, at low area overhead in the single synapse. We use this chip as example implementation for the analyses in Sections 3.4 and 3.5.

All the above architectures are configurable to implement multiple networks after fabrication. This configurability is realized in different forms. The crossbar architecture implements all-to-all connectivity by default, but arbitrary other connectivity can be realized by switching synapses off. However, these synapses are not utilized in this case, so that this architecture may become inefficient at low connection densities, where only a small fraction of the synapses is actually used for a specific network. In the fully addressable matrix, individual synapses are directly addressed from the input pulse packets, so that all connectivity information is stored externally. Switching off synapses is not required in this case; unused synapses simply do not receive input pulses. The other synapse matrix architectures introduced above store part of the routing information in single synapses, for example a part of the source selection. Additionally, synapses may be switched off, while the remainder of the source selection is realized outside of the synapse matrix.

Each of the introduced architectures can be designed such that, after fabrication, an arbitrary set of networks may be configured on it. Then, the question arises, which architecture is the most efficient for a certain set of networks. In order to tackle this question, we first introduce a generalized architecture description that allows to investigate all the above architectures within the same framework.

### 2.3. Generalized architecture

As basis for a more systematic architecture design, a general architecture description is required that can be easily fitted to different connectivity structures, and that contains existing architectures as special cases. Additionally, it should assist area-efficient implementations, maximally sharing circuit components among synapses, and it should exhibit a regular structure for easing layout design.

**Figure 3:**
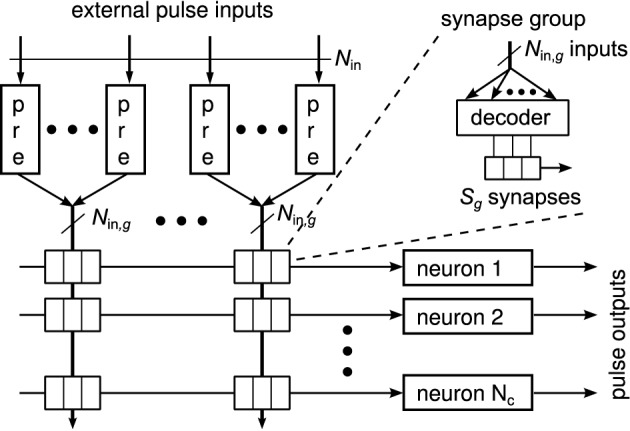
**Generalized matrix architecture, consisting of groups with equal structure**. Each group contains *N*_in, *g*_ pre-synaptic circuits that are connected to *S*_*g*_ synapse columns. Forwarding of inputs to synapses is done by decoders, where each group has its own decoder for each neuron. This arrangement is motivated by the potential to describe different architectures in the same way. Practical implementations would likely use other arrangements, which, however, could still be described in an abstract way using the above architecture.

Figure 3 shows a generic synapse matrix architecture that fulfills these requirements (Partzsch, 2014). Inputs and synapses are divided into equally sized groups, with each group having *N*_in, *g*_ inputs. Each input is fed into a separate circuit block that realizes the pre-synaptic part of the synapse model (cf. Section 2.1). A fixed number of *S*_*g*_ synapses is used per group for each neuron. Connected to these synapses is a decoder that chooses a maximum count of *S*_*g*_ from the *N*_in, *g*_ available inputs of the group. Please note that in an actual implementation, a better choice may be to use one decoder per synapse. However, from a connectivity point of view, this would add redundancy, as multiple synapses could be configured for one input, realizing one synaptic connection multiple times. Each of the *S*_*g*_ synapses implements the pre/post part of the synapse model. It is connected to one neuron, which in turn contains the post-synaptic part of the synapse and the neuron model.

The number of groups *N*_*g*_ determines how many synapses per neuron are implemented, *S* = *N*_*g*_·*S*_*g*_, and how many inputs the block can handle, *N*_in_ = *N*_*g*_·*N*_in, *g*_. The ratio of synapses per input, *S*_*g*_∕*N*_in, *g*_, indicates the typical connection density for which the architecture is designed. Arbitrary higher connection densities can be realized with the architecture by feeding the same input into several groups or by not using some of the inputs of a group. This, however, reduces the number of unique inputs fed into the block.

Each combination of *S*_*g*_ and *N*_in, *g*_ results in a different architecture that can be scaled by the number of groups *N*_*g*_. The existing architectures described in Section 2.2 can be regarded as special cases of the generalized architecture. The crossbar is represented by the values *N*_in, *g*_ = *S*_*g*_ = 1, which makes the synapse decoder obsolete in this case. The fully addressable matrix is effectively generated by having one group of size *N*_in, *g*_ = *S*·*N*_*c*_ and *S*_*g*_ = *S*. With this setting, each synapse can be fed with an individual input. As the number of inputs equals the number of synapses, sharing of pre-synaptic circuits becomes obsolete in this case. The waferscale neuromorphic system of Schemmel et al. (2010) is represented by the values *N*_in, *g*_ = 16 and *S*_*g*_ = 1, as each synapse can choose from 16 inputs. The special feature of connecting neighboring neurons effectively allows to vary *S*_*g*_ between neurons. The architecture of the MAPLE chip constitutes the values *N*_in, *g*_ = 2 and *S*_*g*_ = 1.

The values of *N*_in, *g*_ and *S*_*g*_ influence how well a connectivity model can be replicated on the neuromorphic architecture. This so-called mapping quality (Brüderle et al., 2011) is evaluated in the next section for the generalized architecture.

### 2.4. Evaluation of mapping quality

A crucial quality measure for a given architecture is how well it can realize certain connectivity models, probably those that it was designed for. Previous works have called this measure *mapping quality* and investigated it for existing system designs (Fieres et al., 2008; Brüderle et al., 2011; Petrovici et al., 2014). Apart from technological criteria like integration density and bandwidth considerations, this is the main quality criterion for an architecture. In this article, we use synapse loss as measure of mapping quality. Synapse loss denotes the number of synaptic connections for a realization of a connectivity model that can not be implemented on the architecture due to missing hardware resources or limitations on configurability. Mapping quality in that sense is dependent on the connectivity model to be realized, the hardware architecture and the algorithms used for neuron placement and connection routing (Brüderle et al., 2011). Here, we want to analyse the mapping quality during architecture design, using it as indicator for selecting suitable values for *N*_in, *g*_ and *S*_*g*_. Thus, we try to minimize the influence of neuron placement and connection routing.

We choose uniform random connectivity as benchmark model, where each possible connection between two neurons exists with constant probability *p*, being independent from other connections. In several aspects, this model is the most challenging one for a hardware realization. It is completely unstructured and thus exhibits the highest entropy, in terms of configuration effort, of all networks with the same connection density (Partzsch and Schüffny, 2011). Furthermore, as all neurons are statistically identical, neuron placement has only limited effect on mapping quality. Placement optimization algorithms may only utilize statistical variations, which diminish with network size.

As a side effect of this, uniform random connectivity allows for analytical calculation of synapse loss, avoiding averaging over a high number of network realizations. With each synaptic connection being statistically identical and independent, it is sufficient to do this calculation for one neuron and one synapse group. The number of synapses *s* that are actually required in a synapse group with *N*_in, *g*_ potential synapses is binomially distributed. We thus denote the probability of having *s* out of *N*_in, *g*_ synapses at connection probability *p* with *B*(*N*_in, *g*_, *p, s*) in the following. If a hardware architecture provides *S*_*g*_ < *N*_in, *g*_ synapses for this group, the expected fraction of synapses that can not be mapped to this architecture, i.e., the expected synapse loss *p*_loss, group_, can be calculated as

(1) $$p_{\text{loss},\text{group}}=\frac{\sum_{s=S_{g}+1}^{N_{\text{in},g}}\left(s-S_{g})\cdot B(N_{\text{in},g},p,s\right)}{N_{\text{in},g}\cdot p}\;,$$

with the expected synapse count *N*_in, *g*_·*p* being used to normalize the result. For crossbar architectures, characterized by the setting *N*_in, *g*_ = *S*_*g*_ = 1, the synapse loss according to this formula is always zero.

The above formula expresses the expected synapse loss inside the matrix. However, synaptic connections may also be unroutable if the number of inputs *N*_in_ to the matrix is lower than the required number of inputs *N*_req_. For uniform random connectivity, *N*_req_ is approximately equal to the total number of neurons in the network, *N*, as discussed in Section 2.5. The expected number of unroutable synapses per neuron in this case is (*N* − *N*_in_) · *p*, resulting in an expected synapse loss *p*_loss, in_ of:

(2) $$\begin{array}{l} p_{loss,in}=\frac{\left(N-N_{in}\right)\cdot p}{N\cdot p}=\frac{N-N_{in}}{N}\;. \end{array}$$

Both loss values can be combined by regarding them as loss probabilities and calculating the probability for the complementary event that a connection is routable:

(3) $$\begin{array}{l} p_{loss}=1-\left(1-p_{loss,group}\right)\cdot \left(1-p_{loss,in}\right)\;. \end{array}$$

The analytical loss values calculated above are valid for one-to-one or random neuron placement and input mapping. For the crossbar architecture and the architecture of the MAPLE chip (cf. Section 2.3), optimal mappings can be calculated for single realizations of uniform random networks, using statistical variations for minimizing synapse loss, as described in Noack et al. (2010). In Sections 3.1 and 3.4, we show results for these cases as well.

### 2.5. Rent's rule

In a neuromorphic system, a single chip is often representing only a partition of the overall network. In this case, the question arises on how many external input connections such a partition needs to provide. This issue may be investigated using Rent's rule, an empirical relation between the size of a system's partition and its number of connections with the remainder of the system, first investigated in digital system design (Landman and Russo, 1971; Christie and Stroobandt, 2000). The rule states a power-law relationship between these two quantities, with a characteristic exponent for different system architectures, called Rent exponent.

In the original definition, each connection between two basic elements across the partition boundary is counted as a separate connection. However, this does not take the fan-out of connections from the same sender into account. If an external source connects to several targets inside one partition, a connection to each target is counted separately, ignoring the more efficient solution of forming only one external connection to the partition and splitting it locally. While fan-out is typically low for most of connections in conventional digital systems and can be treated by approximation techniques (Stroobandt and Kurdahi, 1998), it has to be taken into account for neural networks. The solution here is to count all connections from the same sender as one external connection, representing a unique input to the partition (Partzsch and Schüffny, 2012).

**Figure 4:**
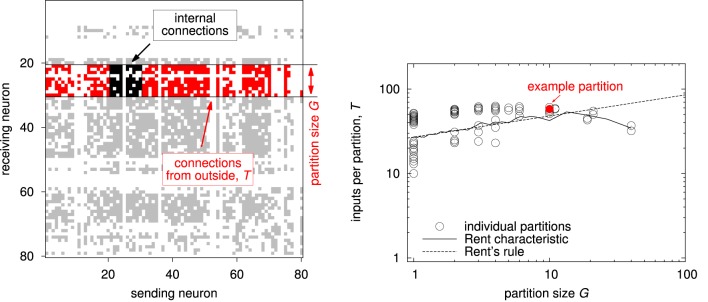
**Generation of Rent's rule and the Rent characteristic. (Left)** Connection matrix of an example network with highlighted single partition. The partition has a size of *G* = 10. Its internal connections are located in a square around the main diagonal, marked in black. All other connections in the same rows have their source outside and their target inside the partition. The number of inputs *T* then is the number of unique outside sources, corresponding to the number of columns with at least one red dot in the figure. **(Right)** Rent characteristic and Rent's rule extracted from a hierarchical partitioning of the network shown in the left plot. The partitioning was created according to Partzsch and Schüffny (2009), using the partitioning algorithm introduced by Hagen et al. (1994). Each point denotes one partition, with the red dot showing the example partition from the plot on the left. The resulting Rent characteristic and Rent's rule are extracted from this data, see text.

Figure 4 illustrates the typical procedure for extracting Rent's rule from a given network (Landman and Russo, 1971; Hagen et al., 1994; Partzsch and Schüffny, 2009; Partzsch, 2014). The network is recursively split into partitions, counting for each of them the number of basic elements *G* (also named partition size) and the number of external inputs *T*, as depicted in the left plot. Values for all partitions are plotted in a log-log diagram of inputs over partition size, each partition representing a single point (see right plot). Fitting a straight line to the data in the logarithmic domain then yields Rent's rule. However, a single power-law relationship may not hold over all partition sizes. This is especially true when counting unique inputs instead of single connections, as the number of inputs is limited by the number of possible senders in the network. Therefore, as an alternative description, averaging over partitions of the same size gives a mean relationship between partition size and number of inputs, which we call Rent characteristic (Partzsch and Schüffny, 2012).

The slope of the Rent characteristic, expressed in the exponent of Rent's rule, determines how the number of inputs scales with the partition size. It can be regarded as a measure of scaling complexity. In geometrical systems, this slope is determined by the system's dimensionality (Bassett et al., 2010).

For some connectivity models, the expected Rent characteristic can be calculated or estimated analytically. For uniform random networks, it increases with maximum possible slope of 1, saturating at the number of senders in the network (Partzsch and Schüffny, 2012). For geometrically localized connectivity, the slope is lower, being 1∕2 in the two-dimensional and 2∕3 in the three-dimensional case, reflecting the dimensionality of the underlying element placement (Landman and Russo, 1971; Bassett et al., 2010).

**Figure 5:**
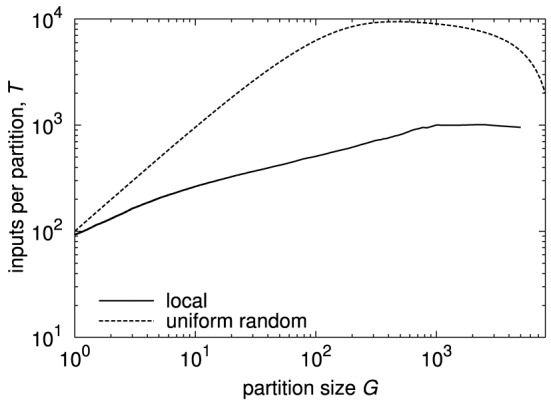
**Rent characteristics for networks with uniform random and local connectivity and 10,000 neurons at approximately the same mean connection density**. Connection probability for uniform random connectivity is *p* = 0.01, Rent characteristic is calculated analytically (see Partzsch and Schüffny, 2012). Connection probability for local connectivity is taken from a Gaussian profile, according to Hellwig (2000), with neurons placed uniformly on a two-dimensional grid, and width of Gaussian scaled to match mean connection density. The Rent characteristic was extracted from one instance, adapting the method described in Partzsch and Schüffny (2009), as the analytical derivation in Partzsch and Schüffny (2012) only results in a rough upper bound.

Figure 5 shows two examples of Rent characteristics, demonstrating the different slopes for uniform random and localized connectivity. For uniform random connectivity, the number of inputs saturates at the number of neurons in the network for a wide range of partitions, starting at a partition size of approximately *G* = 1∕*p*. The negative slope of the curve at big partitions is a side effect of the partition size itself, due to the decreasing number of possible senders outside the partition in this case. The effect of the different slopes is clearly visible from the diagram. While for single elements, i.e., partition size 1, both networks have roughly the same number of inputs, the difference in the number of inputs soon grows to more than an order of magnitude. In a hardware realization, these inputs must be transmitted and handled. Thus, it is likely that the requirements on throughput and address space would differ by an order of magnitude as well in this case.

As introduced in Partzsch and Schüffny (2012), when input counts to partitions of a technical or biological system are known for different partition sizes, these values can be compiled into a limiting Rent characteristic. This characteristic states for each partition size the maximum number of inputs that the system can handle. Thus, comparing the Rent characteristic of a network to the limiting Rent characteristic indicates whether the network fits into the system. If the network's Rent characteristic exceeds the limit, some of the synaptic connections are definitely lost when mapping the network onto the system.

In the next sections, we show limiting Rent characteristics for different neuromorphic architectures, and we describe how to utilize the Rent characteristic for designing efficient neuromorphic architectures. For the reader's orientation, Table 1 summarizes the main symbols used throughout the article.

**Table 1:** **Main symbols used in the article**.

**Symbol**	**Meaning**
*A*	Silicon area
*G*	Size of a system partition
*N*	Number of neurons
*N*_*c*_	Number of neurons per chip
*N*_*g*_	Number of groups in generalized matrix
*N*_in_	Number of inputs
*p*	Connection probability
*p*_loss_	Relative synapse loss
*S*	Number of synapses per neuron
*S*_*g*_	Synapses per group in generalized matrix
*T*	Number of inputs per partition

## 3. Results

### 3.1. Characterization of common neuromorphic architectures

Each of the different state-of-the-art architecture (for an overview, see Section 2.2) results in different restrictions on connectivity, which can be conveniently visualized in their Rent characteristics. In the following, we do this for the two most common synapse matrix architectures, the crossbar and the fully addressable matrix. The main difference between the two is that for the fully addressable matrix, each input drives a single synapse, whereas for the crossbar, it drives a complete synapse column (see also Section 2.2).

As a minimal example for visualizing the impact of the architecture on the realizable connectivity, we use a uniform random network of 200 neurons that are mapped onto two synapse matrices with each 100 neurons and 100 synapses per neuron.

**Figure 6:**
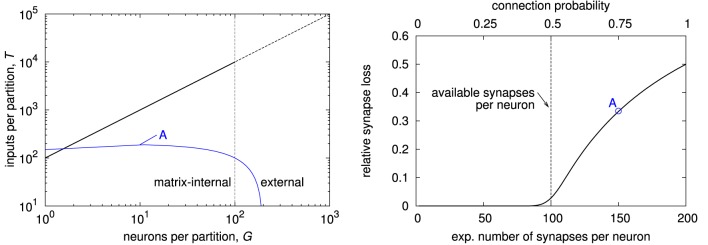
**Characterization of fully addressable synapse matrix. (Left)** Rent characteristic of a fully addressable matrix with *S* = 100 synapses per neuron (black line), and expected Rent characteristic of a uniform random network with *N* = 200 neurons and connection probability *p* = 0.75 (blue line). The network's Rent characteristic exceeds that of the fully addressable matrix, indicating synapse loss. This is because the expected number of synapses *p*·*N* = 150 is too high (left end of Rent characteristic). **(Right)** expected synapse loss with respect to mean number of synapses per neuron for the example network with 200 neurons, equally distributed on two chips, and uniform random connectivity. Label “A” denotes the network whose Rent characteristic is shown in the left plot, resulting in a synapse loss of approximately 34%.

The fully addressable matrix allows to feed each individual synapse with a different input. As a consequence, the number of possible inputs to a partition of neurons grows linearly with the partition size. This results in a Rent characteristic with the maximum possible slope of 1, as shown in the left plot of Figure 6. Thus, the Rent characteristic restricts a network essentially only at its starting point, i.e., at single-neuron partitions (see blue line). In other words, the connectivity is only restricted by the number of synapses per neuron that are provided in the matrix. Consequently in the example network, when increasing connection probability, the relative synapse loss (cf. Section 2.4) increases steadily when the expected number of synapses per neuron in the network exceeds those available in the matrix.

**Figure 7:**
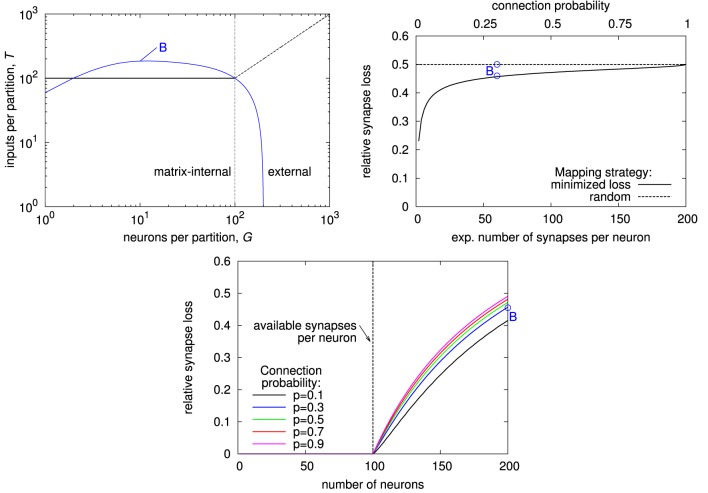
**Characterization of crossbar synapse matrix. (Top left)** Rent characteristic of a crossbar with *S* = 100 synapses per neuron (black line), and expected Rent characteristic of a uniform random network with *N* = 200 neurons and connection probability *p* = 0.3. Like for the example in Figure 6, the network's Rent characteristic exceeds that of the crossbar, caused by the number of synapses per neuron in the crossbar being smaller than the number of neurons in the network (visible as saturation level of the blue curve). **(Top right)** expected synapse loss with respect to mean number of synapses per neuron, for random and optimized neuron-to-input mapping (see text for details). **(Bottom)** expected synapse loss over network size. Label “B” in the top right and bottom plots denotes the network whose Rent characteristic is shown in the top left plot.

In a crossbar architecture, the inputs that are available to a single neuron are the same as the inputs to the whole matrix. In effect, the Rent characteristic stays constant for partition sizes up to the number of neurons in the matrix, as shown in the top left plot in Figure 7. Its further progression depends on how the single synapse matrices are connected. Because Rent characteristics of networks typically increase constantly with partition size, the most restrictive point of the crossbar's Rent characteristic is usually at a whole-matrix partition. This also means that the Rent characteristic and thus the architecture becomes more restrictive when increasing the number of neurons in the matrix. In our example, the number of inputs to the matrix, equalling the number of synapses per neuron, is only half the number of neurons, so that the Rent characteristic of the uniform random network exceeds that of the crossbar (see blue line in the top left plot). Therefore, when mapping a realization of the network onto the architecture, expected synapse loss is 0.5, irrespective of the connection probability, as shown in the top right plot in Figure 7. For a single realization, synapse loss may be minimized by choosing from the sender neurons those 100 that form the most synapses with the neurons placed on the matrix. This reduces synapse loss for low connection probabilities, but not to an acceptable level. In essence, uniform random connectivity can only be faithfully mapped onto crossbar architectures as long as the network size does not exceed the number of synapses per neuron. The lower plot of Figure 7 illustrates this: Synapse loss sharply increases with network size once that size reaches the number of synapses per neuron in the matrix, with only minor dependence on connection probability.

The above analysis introduced the Rent characteristic as a tool for verifying compatibility of an architecture with a network model. The next section shows how to utilize the Rent characteristic during system design.

### 3.2. Neuromorphic system design

Our approach to neuromorphic system design starts from a given connectivity model, deriving the system architecture and its basic specifications from it. This is done in two steps, following a top-down approach. First, the system hierarchy is fixed and basic numbers for single modules derived. Second, the architecture of a single chip or block is designed.

The design of the system hierarchy is dependent on a multitude of factors, with connectivity being only one of them. The granularity of the hierarchy, i.e., the number of sub-modules on each hierarchy level, is therefore a trade-off to be defined at the beginning of the design process. Once these numbers are defined, the number of required connections between sub-modules on different levels of the hierarchy may be derived with the Rent characteristic.

For this, a Rent characteristic needs to be defined that covers the classes of networks that are to be implemented on the hardware. The Rent characteristic of a single network typically constitutes a mean over all partitions, cf. Section 2.5. In contrast, a Rent characteristic used for system design has to cover variations between partitions as well, so that some safety margin may have to be added.

The Rent characteristic *T*(*G*) directly relates to the basic numbers of a single chip that are described in Section 2.2, i.e., the number of neurons *N*_*c*_, the number of synapses per neuron *S* and the number of external inputs to the chip *N*_in_. The number of synapses per neuron corresponds to the number of inputs to a single-neuron partition, i.e., *S* = *T*(1). The number of neurons per chip *N*_*c*_ is not only defined by the connectivity, but also depends on the total chip area and the circuit areas per neuron and synapse. Once this number has been chosen, the required number of inputs to the chip can be extracted from the Rent characteristic at the partition size *N*_c_: *N*_in_ = *T*(*N*_c_).

**Figure 8:**
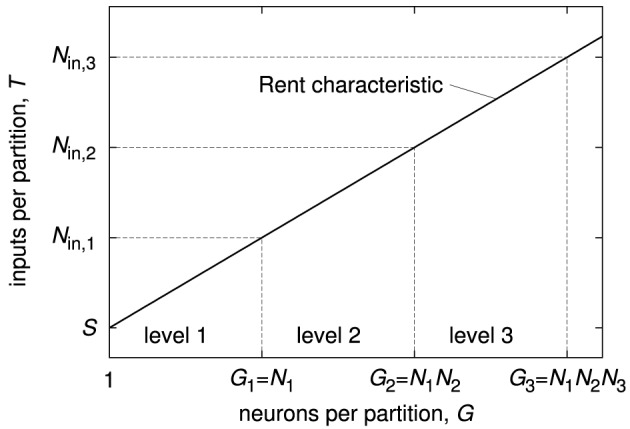
**Relation of the Rent characteristic and basic numbers of a hierarchical neuromorphic system with three hierarchy levels**. As example Rent characteristic, a single power law was chosen. *N*_1_ denotes the number of neurons inside one level-1 module, *N*_2_ the number of level-1 modules inside one level-2 module, and *N*_3_ the number of level-2 modules inside one level-3 module. *N*_in, 1_, *N*_in, 2_, and *N*_in, 3_ are the number of inputs of one module on level 1, 2, and 3.

The same relation is present also on higher levels of the system hierarchy, as shown in Figure 8. In general, the number of inputs on each level equals the value of the Rent characteristic at the partition size of the total number of neurons inside that level. In other words, if the number of neurons on level 1 is *N*_1_ and the number of level-1 modules inside a level-2 module is *N*_2_, the number of inputs for a level-1 module is *T*(*N*_1_), whereas it is *T*(*N*_1_·*N*_2_) for a level-2 module.

**Figure 9:**
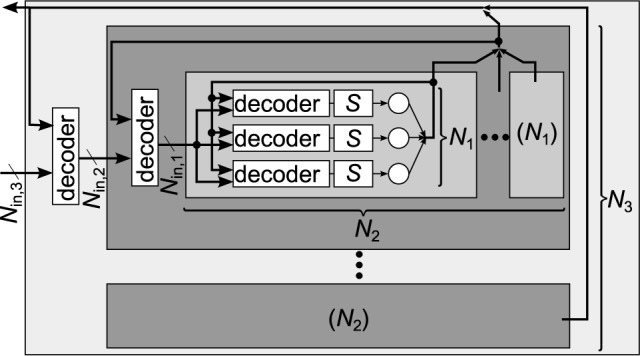
**Generic connectivity structure of a hierarchical neuromorphic system with three hierarchy levels, as it would be constructed from the Rent characteristic in Figure 8 (Partzsch, 2014)**. Single neurons are depicted as circles and all *S* synapses of a neuron are joined in one block. On each level, a module contains a number of sub-modules and one decoder per sub-module, which chooses the inputs of the sub-module from all sources available to the module. Available sources are the external inputs and the outputs of all neurons inside the module. On the lowest level, this resembles the generic chip structure shown in Figure 2.

Figure 9 shows an abstract structure of a hierarchical neuromorphic system that is defined only by the numbers in Figure 8. On each hierarchy level, inputs from outside and outputs of all neurons from inside the level form the set of connection sources that have to be distributed to the sub-modules. As the number of sources is typically bigger than the number of inputs to one sub-module, a decoder is needed for each sub-module, choosing the inputs from the available sources. A single neuron-to-neuron connection passes one or multiple decoders, dependent on the location of the neurons in the system. The set of all decoders defines the available configuration space for connectivity, which can be quantified as minimally required configuration memory (Partzsch and Schüffny, 2011). If the decoders do not have any further restrictions, i.e., they can be configured to choose any subset of their inputs, the Rent characteristic completely represents the restrictions on connectivity: Any network can be realized with the architecture, as long as input counts of the network on all partition sizes do not exceed the architecture's Rent characteristic.

### 3.3. Design of the synapse matrix architecture

Having defined the basic numbers of a neuromorphic chip according to Section 3.2, i.e., knowing the number of neurons *N*_*c*_, the number of synapses per neuron *S* and the number of inputs to the chip *N*_in_, the question arises on how to design a synapse matrix architecture that is fitted to these numbers. The generalized matrix architecture (see Section 2.3) can be utilized for this task. The basic building block of this architecture is one group with *N*_in, *g*_ inputs and *S*_*g*_ synapses per neuron. With *N*_*g*_ groups in the matrix, the total number of synapses per neuron and the number of inputs calculate as *S* = *N*_*g*_·*S*_*g*_ and *N*_in_ = *N*_*g*_·*N*_in, *g*_. Thus, in principle, the synapse-to-input ratios of group and chip equalize, *S*∕*N*_in_ = *S*_*g*_∕*N*_in, *g*_. This ratio can be regarded as local synapse density, i.e., the fraction of available connections from all possible input-to-neuron connections.

For deriving suitable values for *N*_in, *g*_ and *S*_*g*_, we make the assumption of uniform random connectivity, i.e., equal probability for all possible input-to-neuron connections (for a motivation of this choice, see Section 2.4). We set the connection probability equal to the synapse density: *p* = *S*∕*N*_in_. For a given group size *N*_in, *g*_, we can then calculate the minimum value for *S*_*g*_, such that networks with the given connection probability *p* can be mapped to the architecture with a certain minimum mapping quality. That is, the expected synapse loss stays below a pre-defined maximum value. For the following results, we use analytical calculations of synapse loss, as introduced in Section 2.4.

**Figure 10:**
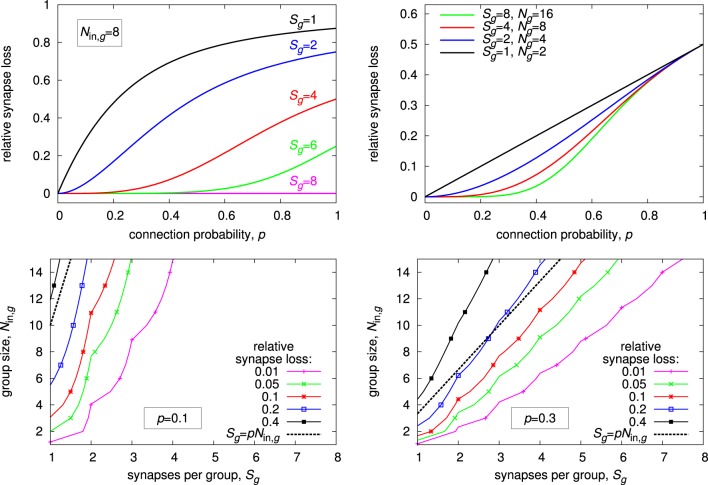
**Expected synapse loss for different group configurations. (Top left)** progression of synapse loss with connection probability for different number of synapses per group at a constant group size of *N*_in, *g*_ = 8. **(Top right)** same as top left, but group size is always twice the number of synapses, *N*_in, *g*_ = 2·*S*_*g*_. **(Bottom row)** isoline plots of synapse loss with respect to group size and synapses per group, for a constant connection probability of *p* = 0.1 **(bottom left)** and *p* = 0.3 **(bottom right)**.

Results for different configurations are summarized in Figure 10. Looking at synapse loss with respect to connection probability (top left and top right plot), there is always a region of probability values for which the synapse loss stays close to zero, except for *S*_*g*_ = 1, where it rises approximately linearly at small connection probabilities. The region of low synapse loss effectively defines the operating regime of the respective architecture. For a fixed synapse-to-input ratio (top right plot), this region extends with increasing number of inputs *N*_in, *g*_. While this speaks in favor of groups with high input count, decoder implementations become more complex with more inputs, counterbalancing this advantage in practice.

For an actual architecture design, connection probability, and maximum acceptable synapse loss are given, and suitable combinations of *N*_in, *g*_ and *S*_*g*_ have to be found under these constraints. The isoline plots in the bottom row of Figure 10 may be utilized for this task. Suitable values for *N*_in, *g*_ and *S*_*g*_ may be taken directly along or below the isoline with the desired maximum synapse loss. In general, choosing a combination with a low synapse-to-input ratio, i.e., a point toward the top left corner in the plots, is preferable, as it results in a comparatively low total number of synapses per neuron *S*, which calculates as *S* = *S*_*g*_·*N*_*g*_ = *S*_*g*_·*N*_in_∕*N*_in, *g*_. The plots also show that a choice according to the expected value, i.e., *S*_*g*_ = *p*·*N*_in, *g*_, results in a relatively high synapse loss of 20% or more. This is a consequence of local statistical variations, requiring an increased synapse count *S*_*g*_ for compensation. The effect is more pronounced at lower connection probabilities.

In the next section, we investigate the special case *N*_in, *g*_ = 2, *S*_*g*_ = 1 in more detail, which yields additional potential for reducing synapse loss.

### 3.4. Example architecture: the MAPLE chip

As an example architecture, we now further investigate the case *N*_*g*_ = 2, *S*_*g*_ = 1, which has been implemented in the MAPLE chip (Mayr et al., 2013). As described in Noack et al. (2010), this parameter choice allows for implementation as an extended crossbar, where input driver circuits are placed on both sides of the synapse matrix and a switch is added to each synapse for choosing between the two input drivers per column. Compared to a crossbar, this retains the advantages of shared input circuits between synapses and simple layout, while doubling the number of inputs to the matrix at no additional synapses.

At first sight, the MAPLE architecture is not a sensible choice, because expected synapse loss increases linearly with connection probability, as shown in the top right plot of Figure 10. However, this architecture allows for explicit calculation of an optimal input-to-group configuration, significantly reducing synapse loss compared to a random placement (Noack et al., 2010). In fact, the MAPLE architecture is the only case of a generalized matrix architecture, except for the crossbar (cf. Section 3.1), for which such an explicit calculation is currently possible. For all other configurations of *N*_*g*_ and *S*_*g*_, improving mapping quality by changing input-to-group configuration has to resort to heuristic methods.

**Figure 11:**
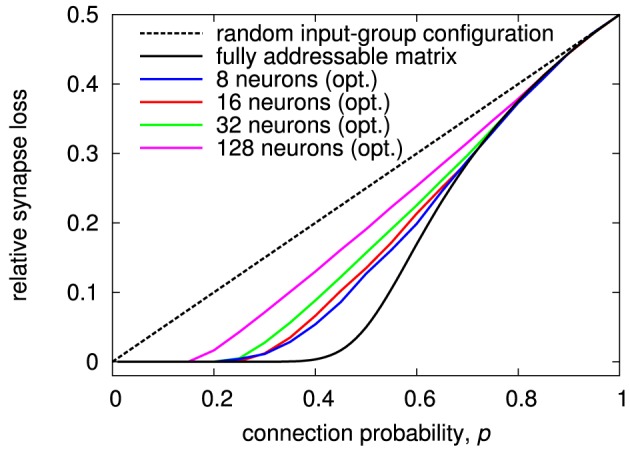
**Synapse loss of generalized architecture with *N*_*g*_ = 2 and *S*_*g*_ = 1 for different number of neurons per matrix with optimized input-to-group configuration**. Like for the examples in Section 3.1, the optimization was performed for a two-chip setup, so that the network size is twice the number of neurons per chip.

Results for the optimized input-to-group configuration are shown in Figure 11. The effectiveness of the optimization is dependent on the number of neurons in the matrix. It is less pronounced at higher neuron count, because the optimization utilizes local statistical variations that are reducing with the number of neurons. The optimized result always exhibits a region with synapse loss close to zero, like for configurations with more inputs and synapses per group (cf. Figure 10). In effect, this optimization makes the MAPLE architecture a simple, yet attractive, alternative to other variants of the generalized matrix architecture.

### 3.5. Area comparison

The considerations so far were concerned with the assessment of mapping quality (i.e., synapse loss) for a given type of network. As a result, each synapse matrix architecture can be designed for ensuring a certain mapping quality, by providing a sufficient number of synapses.

However, the final goal of a system design is to arrive at an efficient architecture, using minimum resources on silicon area and power (Hasler and Marr, 2013). As a first step in investigating efficiency, we provide an area comparison for the architectures analyzed above.

For calculating the total area of an architecture, circuit areas of the single components have to be known. Following Figure 1, we separate here between the individual synapse circuit (pre/post part), occupying area *A*_syn_, and the pre-synaptic part with *A*_pre_, which can be shared between synapses having the same pre-synaptic input. The investigated architectures do not differ in the arrangement of the post-synaptic part and the neuron circuit. Therefore, we leave these parts out for the area comparison. For all variants of the generalized matrix architecture except crossbar and fully addressable matrix, individual synapses need to be extended by a decoder for choosing one input from their group. We denote the corresponding additional area per synapse as *A*_dec_.

The architectures mainly differ in the total number of synapses that are required to achieve a certain mapping quality. For the following example, we require relative synapse loss to be below 5%. Then, for the crossbar architecture, the number of synapses per neuron has to be the same as the number of inputs: *S*_cb_ = *N*_in_, cf. Section 3.1. In principle, the number could be reduced by 5%, but this would not save much. Due to more configurability, the synapse count typically is smaller for other choices of the generalized architectures, and can be extracted from isoline plots as those in Figure 10. We denote it as *S*_gen_ in the following. As a special case, the fully addressable matrix is only constrained by the number of synapses per neuron. The required synapse count *S*_fa_ can be directly derived from the synapse count distribution of the network, which is binomial for uniform random connectivity.

The total areas for the crossbar, fully addressable matrix and generalized matrix architecture then calculate as follows:

(4) $$\begin{array}{lllll} crossbar:\; & A_{cb} & =N_{in}\cdot N_{c}\cdot A_{syn}+N_{in}\cdot A_{pre}\; & \; & \; \end{array}$$

(5) $$\begin{array}{lllll} fullyaddr.:\; & A_{fa} & =S_{fa}\cdot N_{c}\cdot \left(A_{syn}+A_{pre}\right)\; & \; & \; \end{array}$$

(6) $$\begin{array}{lllll} generalized:\; & A_{gen} & =S_{gen}\cdot N_{c}\cdot \left(A_{syn}+A_{dec}\right)+N_{in}\cdot A_{pre}\; & \; & \; \end{array}$$

For better comparability between different implementations, all areas may be normalized by the area of a single synapse. Then, the remaining area contributions are the relative pre-synaptic area *A*_pre_∕*A*_syn_ and the relative decoder area *A*_dec_∕*A*_syn_.

As an illustrative example, we take the same network as in Section 3.1, i.e., 200 neurons divided evenly onto two chips, and fix the connection probability at *p* = 0.1. Due to the uniform random connectivity, the number of inputs per chip equals the number of neurons, *N*_in_ = 200. For the fully addressable matrix, a synapse count of *S*_fa_ = 27 is required for achieving less than 5% relative synapse loss. As example of the generalized matrix architecture, we use the MAPLE architecture, whose mapping quality is sufficient for the given network as well, cf. Figure 11. Thus, we set *S*_gen_ = 100. With these synapse counts, areas can be calculated and compared dependent on the relative sizes of pre-synaptic and decoder circuit.

**Figure 12:**
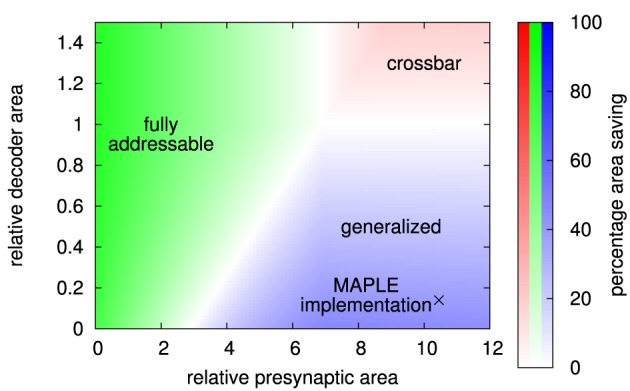
**Area comparison for crossbar, fully addressable matrix, and MAPLE (i.e., generalized) architecture, implementing the example network**. Color indicates the best architecture, while color intensity denotes percentage area saving compared to the next-best architecture.

Results of this calculation are shown in Figure 12. A fully addressable matrix architecture is most area-efficient if the pre-synaptic circuit is small. Then, sharing of pre-synaptic circuits between synapses, as done by the other architectures, has only little area advantage. In contrast, minimizing the number of synapse circuits is crucial in this case. The crossbar architecture is most efficient if the pre-synaptic circuit is comparatively big and decoders in individual synapses would cause a significant area overhead. In other words, a single synapse circuit is cheap in terms of area. This is the case for memristive devices, where driver circuits have to contain the complete synaptic waveform generation (Zamarreno-Ramos et al., 2011; Mayr et al., 2012; Saighi et al., 2015) and integration of decoders in synapses would result in a high area penalty.

The generalized matrix architecture is best for big pre-synaptic circuits, but small area overhead for synaptic decoders. This is the typical case for CMOS implementations that include short-term plasticity or implement long-term plasticity rules that allow to move parts of the plasticity calculation to the pre-synaptic circuit. The MAPLE chip is a typical example of this case (Noack et al., 2010; Mayr et al., 2013). Its core synapse circuits require a silicon area of 660 μm^2^ each. With decoder, a synapse occupies 750 μm^2^, resulting in relative decoder overhead of 14%. The size of the pre-synaptic circuit, including pre-synaptic waveform generation for the local correlation plasticity rule (Mayr and Partzsch, 2010), takes 6900 μm^2^ silicon area, which is a factor of 10.5 bigger than the core synapse circuit. In Figure 12, this point lies deep inside the area where the generalized matrix is most area-efficient. Thus, the MAPLE architecture is indeed the most area-efficient choice in this case.

In summary, a comparing diagram such as Figure 12 can be generated from dimensioning the different architectures according to a given network. It is thus independent of technology or circuit design. However, together with rough estimates on circuit sizes, it allows for an informed decision on the most area-efficient architecture.

## 4. Discussion

In this article, we have introduced methods for analysing neuromorphic hardware architectures, building on previous work (Partzsch and Schüffny, 2011, 2012; Partzsch, 2014). We have also shown how to systematically design architectures from a pre-defined network model, and demonstrated how to find the most area-efficient architecture for a given use case.

Using the Rent characteristic for summarizing the connectivity of a neuromorphic system is a useful tool, being relatively simple to derive, yet highly descriptive when comparing to concrete network examples. It can be used as a benchmark indicator, comparing performance of neuromorphic systems in emulating synaptic connectivity. It furthermore allows to start the system design from a network model, and can be utilized in major design decisions. This enables a network-driven design process, which is an important advantage compared to previous, trial-and-error based works on architecture design (Fieres et al., 2008; Navaridas et al., 2009). Along these lines, we also introduced a generalized matrix architecture that is a practical tool for analysing and designing neuromorphic architectures with a synapse matrix.

While the general, network-driven design approach is applicable to a wide range of network structures, our investigations in this paper were restricted to uniform random connectivity. We chose this model because of simplicity and the possibility for analytical calculations. Other connectivity models could principally be characterized by connection density changing over the network. In the Rent characteristic, this may result in partitions of the same size with highly variable number of inputs. That would require calculating a maximum Rent characteristic that covers all partitions, instead of using the mean and some safety margin to account for statistical variations. However, if changes in connection density are caused by some underlying geometrical restrictions, the Rent characteristic is likely to cover them well (Partzsch and Schüffny, 2012), allowing to employ them in the system design.

In the generalized matrix architecture, an increased local connection density can be supported by feeding one external input to several groups, at the expense of less different inputs. The same can be done neuron-wise by connecting two or more neuron circuits together to form a single neuron with more synapses, as already employed by Schemmel et al. (2010) and Qiao et al. (2015). As a result, arbitrary fan-in and fan-out distributions could be realized, following a similar approach as for generalized random graphs (Chung and Lu, 2002). In effect, a design fitted for low connection density and reserves in the number of inputs allows for adaptation to locally changing connection densities. However, this approach does not necessarily capture specific, non-random connectivity, like nearest-neighbor connections, which are better implemented with specialized architectures (Choi et al., 2004).

While our investigations on synapse matrix architectures have no straightforward link to other implementation approaches, some of the methods can be utilized to characterize connectivity constraints in these systems as well. Multi-synapses, implementing one synapse circuit for multiple synaptic connections with superimposing activation functions (Vogelstein et al., 2007; Benjamin et al., 2014), pose no hard limits on synapse count and network architecture on chip level. However, they are limited by the input bandwidth, which can be analyzed with the Rent characteristic, given some mean spiking activity per connection. The same is true for inter-core and inter-chip bandwidth in multi-processor systems like SpiNNaker (Furber et al., 2014). Dimensioning of the routing resources in FPNAs (Farquhar et al., 2006) may utilize the Rent characteristic as well.

Our approach explicitly evaluates architectures for their mapping quality, expressed as synapse loss when realizing a certain network on the architecture. Synapse loss causes deviations in connectivity, which in turn may have consequences on network behavior, as investigated for example by Brüderle et al. (2011) and Petrovici et al. (2014). Dependent on the cause of the synapse loss, network dynamics may be affected differently. If the maximum available number of synapses per neuron is too low, neurons receive less input than expected. This effect is strongest for those neurons with the most synapses. As a consequence, both the mean of spiking activity and its variance between neurons may reduce. If the number of inputs to a group of neurons is restricted, for example due to a limited number of inputs per chip, connections from additionally required source neurons can not be realized. This reduces the variety of inputs and may result in more correlated activity. How these deviations affect the overall performance of a network has to be analyzed individually. Results of this investigation can in turn be utilized in architecture design, because a higher tolerable synapse loss often reduces the number of synapses to be implemented, as our results show.

In terms of resource efficiency, we have limited our investigations in this article to silicon area. However, architectural choices also have great impact on energy efficiency. The crossbar and generalized matrix architectures inherently allow for sending one input event to several target synapses, realizing part of the axonal fan-out. In contrast, in the limit case of a fully addressable matrix, one input event stimulates one individual synapse, so that the axonal fan-out has to be performed completely off-chip. In other words, the same spike event has to be transmitted several times to form all desired connections with the neurons on the chip. As a result, both required input bandwidth and total energy per event distribution are multiplied. The same applies to multi-synapse architectures. This example shows that a suited architecture design is also vital for pushing neuromorphic systems to better energy efficiency (Hasler and Marr, 2013), which is both the main promise and challenge of neuromorphic engineering.

### Conflict of interest statement

The authors declare that the research was conducted in the absence of any commercial or financial relationships that could be construed as a potential conflict of interest.
